# Bioactive Compounds from Marine-Derived *Aspergillus*, *Penicillium*, *Talaromyces* and *Trichoderma* Species

**DOI:** 10.3390/md16110408

**Published:** 2018-10-26

**Authors:** Rosario Nicoletti, Francesco Vinale

**Affiliations:** 1 Council for Agricultural Research and Agricultural Economy Analysis, OFA Research Centre, 81100 Caserta, Italy; 2 Institute for Sustainable Plant Protection, National Research Council, 80055 Portici (NA), Italy; francesco.vinale@ipsp.cnr.it

The impact of bioactive compounds from natural sources on human life, particularly in pharmacology and biotechnology, has challenged the scientific community to explore new environmental contexts and the associated microbial diversity. As the largest frontier in biological discovery, the sea represents one of the most conducive reservoirs of organisms producing secondary metabolites with interesting biological activities. In the last decades fungi have received increasing attention, both for their pervasive occurrence in several habitats and for their widespread aptitude to develop symbiotic associations with higher organisms. In many cases, fungal strains have been reported as the real producers of drugs that were previously ascribed to marine plants and animals [1,2].

Species of the genera *Aspergillus*, *Penicillium*, *Talaromyces* and *Trichoderma* are renowned producers of bioactive compounds [3,4,5]. Until recently they were considered as ‘terrestrial’ fungi with merely accidental discoveries in marine environments. However, recent findings have demonstrated that actually they are very abundant in marine environments and sometimes establish symbiotic interactions with higher organisms (e.g., the case of *Aspergillus sydowii* on gorgonians [6]). It can be assumed that many species belonging to these genera of Ascomycetes are rather eclectic in their ability to adapt and thrive in very different environmental conditions. Thus, at least in terms of species number, *Aspergillus* and *Penicillium* respectively represent the first and the second most abundant genera of filamentous fungi reported from marine contexts [4,7].

Papers included in this special issue deal with marine-derived species of *Aspergillus*, *Penicillium*, *Talaromyces* and *Trichoderma*, providing a good overview of their biosynthetic potential. New compounds have been isolated and characterized from strains of *A. candidus* [8], *A. clavatus* [9], *A. tritici* [10], *P. raistrickii* [11], and *Talaromyces purpurogenus* [12]. Two papers report the recovery of strains of *Aspergillus* and *Penicillium* [13,14] that could not be ascribed to known species, thus underlying that new findings from the marine environment can expand the current taxonomic diversity and eventually contribute to a more coherent classification. Moreover, data concerning several known fungal compounds were discussed, providing clues for a better comprehension of their biosynthetic processes, and a useful indication for chemotaxonomy. Taxonomic implications and their relevance for a correct integration of new data in the current knowledge have been also discussed in a review on mangliculous strains of *Talaromyces*, after the recent separation of this genus from *Penicillium* [15].

Several novel compounds characterized from the culture filtrates of these fungi present some original or uncommon structures, such as: the raistrickiones, that represent the first case of 3,5-dihydroxy-4-methylbenzoyl derivatives of natural products [11]; the indole-diterpene alkaloids asperindoles C and D, containing a 2-hydroxyisobutyric acid residue [13]; 9,10-diolhinokiic acid, which is the first thujopsene-type sesquiterpenoid containing a 9,10-diol moiety, and roussoellol C containing a novel tetracyclic fusicoccane framework [12].

Significantly, most of the above compounds displayed biological activity as radical-scavengers [11], inhibitors of isocitrate dehydrogenase [14], antibiotic and/or cytotoxic agents. Antibiosis ranged from the antifungal activity of the coumarin, chromone, and sterone derivatives produced by *A. clavatus* R7 [9], to the antibacterial effects exhibited by several compounds towards methicillin-resistant strains of *Staphylococcus aureus*, vancomycin-resistant strains of *Enterococcus faecalis*, and *Vibrio* spp. [8,10]. The assays carried out on several human tumor cell lines were indicative of general antiproliferative effects [8,10,12,13,14]. In the case of penitrem A, a previously known mycotoxin, a potential for its use in cancer therapy was disclosed, based on the BK channel affinity and other side effects, which characterize this product as a possible novel sensitizing and chemotherapeutic synergizing agent [16].

In conclusion, we are grateful to all authors who contributed to our Special Issue, in the expectation that at least part of their work may have a follow up with new and exciting discoveries.
